# One-year visual and anatomical outcomes of intravitreal faricimab injection for neovascular age-related macular degeneration after prior brolucizumab treatment

**DOI:** 10.1038/s41598-024-59894-8

**Published:** 2024-04-20

**Authors:** Hironori Takahashi, Satoru Inoda, Hidenori Takahashi, Ryota Takahashi, Yuto Hashimoto, Hana Yoshida, Hidetoshi Kawashima, Yasuo Yanagi

**Affiliations:** 1 Department of Ophthalmology, Jichi Medical University, 3311-1 Yakushiji, Shimotsuke-shi, Tochigi 329-0431 Japan; 2 Department of Ophthalmology and Micro-Technology, Yokohama City University, Yokohama, Japan; 3 Retina Research Group, Singapore Eye Research Institute, Singapore Eye-ACP, Duke-NUS Medical School, National University of Singapore, Singapore, Singapore

**Keywords:** Brolucizumab, Age-related macular degeneration, Faricimab, Switching, Retinal diseases, Outcomes research

## Abstract

This single-center retrospective cohort study analyzed the 1-year real-world treatment outcomes of 63 consecutive eyes (of 60 patients) with neovascular age-related macular degeneration (nAMD) that were switched from intravitreal brolucizumab (IVBr) to intravitreal faricimab (IVF) and managed on a treat-and-extend regimen with discontinuation criteria. After the switch, patients opted to continue IVF, to switch back to IVBr, or receive photodynamic therapy (PDT). Thirty-eight patients continued IVF, 16 patients were switched back to IVBr, 2 patients received PDT, and 4 patients paused treatment. Best-corrected visual acuity (BCVA), central subfield thickness (CST), subfoveal choroidal thickness (sf-CT), and injection intervals were compared immediately before and 1 year after the initial IVF. Whereas there was no change in BCVA and CST; 0 [− 0.0969 to 0.125, *P* = 0.58], − 1.5 [− 27.8 to 13.5, *P* = 0.11] µm, respectively, sf-CT decreased significantly; − 19.5 [− 45.5 to 7.75, *P* = 0.015] µm. The patients switched back showed no significant change in sf-CT. The injection interval extended significantly in the IVF continuation and the switch-back group (2.0 and 3.0 weeks, respectively; [*P* = 0.0007 and 0.0078]) in eyes with a pre-switching interval of less than 12 weeks. Faricimab shows promise as a safe and effective alternative to brolucizumab for treating nAMD.

## Introduction

Intravitreal anti-vascular endothelial growth factor (VEGF) injections are the first-line therapy for neovascular age-related macular degeneration (nAMD)^1^. Brolucizumab was first approved by the United States Food and Drug Administration and then by the relevant authorities in various countries, based on HAWK and HARRIER trials^2^. Reports of its efficacy in the real-world setting have shown relatively favorable results compared with aflibercept for treating subretinal pigment epithelial lesions and reducing choroidal thickness^3–7^. However, several post-marketing studies have reported higher than expected rates of intraocular inflammation following intravitreal brolucizumab (IVBr) injection, including cases of occlusive retinal vasculitis^8–10^, prompting increased clinical caution surrounding the use of IVBr.

Faricimab, which targets not only VEGF but also angiopoietin (Ang)-2, was subsequently approved in 2022 by the United States Food and Drug Administration (FDA) and regulatory agencies in various countries^11,12^. The TENAYA and LUCENE trials showed that the effect of faricimab appears to be prolonged compared with that of aflibercept in terms of maintaining best-corrected visual acuity (BCVA) outcomes^13^. Following the introduction of faricimab, short-term studies reported improved BCVA and retinal morphology during the loading phase of intravitreal faricimab (IVF) treatment in patients with treatment-naïve nAMD^14,15^. A few studies have shown efficacy of IVF in nAMD patients who have shown resistance to aflibercept and/or bevacizumab^11,16^. However, there is a lack of long-term data on clinical outcomes after switching to IVF from other anti-VEGF agents.

With respect to switching from IVBr injections, we previously evaluated the safety and efficacy of a single IVF injection in eyes with nAMD which were previously treated with IVBr^17^. The present study is an extension of this previous study and aimed to investigate the longer term treatment outcomes of IVF 1-year after switching from IVBr.

## Methods

### Ethical approval and consent

The study had a single-center retrospective design and was approved by the institutional review board of Jichi Medical University (JICHI20-127) and adhered to the tenets of the 1964 Declaration of Helsinki and its later amendments or comparable ethical standards. The study procedures followed our institutional guidelines, and all patients provided oral informed consent before the procedures were performed.

### Procedure

Consecutive patients who undergoing treatment with brolucizumab were provided comprehensive drug information regarding faricimab, and patients who voluntarily opted to switch to faricimab were included in this observational study. Between May 2022 and September 2022, among 225 patients who were treated with brolucizumab, 60 chose to switch to faricimab. A total of 63 eyes of 60 patients with nAMD previously treated with brolucizumab were enrolled in this study. Patient demographic and clinical characteristics are shown in Supplementary Table S4.

MNV was diagnosed based on multimodal imaging including fundus examination, fundus photography, fluorescein angiography (FA) and indocyanine green angiography (ICGA), or swept-source optical coherence tomography (OCT; performed by HT, SI, or RT)^17^. FA and ICGA were performed before the first injection of the previous anti-VEGF treatment. Color fundus photographs were obtained using a commercially available fundus camera system (VX-10; Kowa Co., Ltd., Nagoya, Japan). FA and ICGA were performed using a confocal scanning laser ophthalmoscope (Heidelberg Retina Angiography; Heidelberg Engineering, Heidelberg, Germany). Cross-sectional images of the macula were obtained by swept-source OCT (DRI OCT Triton; Topcon, Tokyo, Japan).

At baseline, defined as immediately before switching to IVF, all patients underwent an ophthalmic assessment with refraction, BCVA testing, slit-lamp biomicroscopy with or without contact lenses, indirect ophthalmoscopy, color fundus photography, and swept-source OCT^17^.

### Treatment regimen

The patients had been in the maintenance phase of a treat-and-extend (TAE) regimen, with the treatment interval being extended up to 16 weeks with adjustment intervals of 2 or 4 weeks prior to switching to IVF. No loading therapy was administered upon switching^17^, and the patients continued the TAE regimen after the initial IVF. The injection interval at switching was instructed to maintain their regular visit intervals (± 1 week) after the initial IVBr injection, regardless of the presence or absence of exudative changes on that day. In this TAE regimen, disease activity was defined as new retinal hemorrhage, presence of fluid on OCT, or change in pigmented retinal detachment. Fluid was defined as the presence of any intraretinal fluid, and any subretinal fluid greater than 100 µm in height at the subfoveal center. Subfoveal subretinal fluid of 100 µm or less or any subretinal fluid elsewhere was tolerated if it appeared without affecting vision, and the treatment period was maintained under these conditions^18–20^. There is no apparent correlation between anatomical and functional improvement in most eyes with PED and nAMD^21,22^. We considered changes in serous PED to be tolerable and upheld the treatment interval with the change of serous PED alone. If an injection interval shorter than the patients’ previous treatment interval was recommended, they had the option to switch back to brolucizumab or receive photodynamic therapy (PDT).

Based on previous reports that visual acuity can be maintained even with the treatment interval of 16 weeks, we set the maximum treatment interval to 16 weeks^23^. In addition, considering studies indicating that recurrence is less likely to occur when dry macula was confirmed at an interval of 16 weeks (rather than 12 weeks), we devised a treatment-free interval protocol^24–30^. Patients with a dry macula for 2 consecutive visits with a maximal treatment interval of 16 weeks were allowed to enter a treatment pause and switch to a pro re nata (PRN) regimen with regular monitoring every 3 months.

### Inclusion and exclusion criteria

This study included patients with at least a 1-year history of treatment with IVBr, and who had been following a TAE regimen and received at least 3 IVBr injections within 1 year prior to switching to IVF. This study excluded patients who experienced a recurrence of nAMD after a stable follow-up period of ≥ 6 months and those who received their first IVF injection as a reactive treatment.

Between May 2022 and September 2022, among the 225 patients who were treated with brolucizumab, 62 patients had a treatment history of less than 1 year and 55 patients were treated with a pro re nata regimen. Among the remaining 108 patients, 15 patients opted to continue treatment with IVBr. Among the 93 patients who decided to switch to faricimab, 33 had a follow-up period of less than 1 year and were excluded, leaving 60 patients with a follow-up period of 1 year of longer for analysis.

Forty eyes of 38 patients (63.3%) continued IVF (IVF continuation group), whereas 16 eyes of 16 patients were switched back to brolucizumab (switch-back group), and 3 eyes of 2 patients received PDT (PDT combination group). Three eyes of 4 patients (6.7%) chose monitoring because of dry macula after a mean of 2 injections (dry macula group).

### Outcome measures

We evaluated changes in BCVA, central subfield thickness (CST), and subfoveal choroidal thickness (sf-CT) 1 year after the initial IVF. Patients were classified into two groups based on the injection interval before the switch, with one group receiving treatment at intervals of less than 12 weeks and the other group receiving treatment at intervals of 12 weeks or more. We then performed a comparative analysis of changes in BCVA, CST, sf-CT, and injection intervals. The post-switch injection interval, occurring 1-year after, was defined as period between injections 1-year after from the first IVF injection. This definition was used to mitigate the impact of the previous treatments on subsequent injections.

In a safety outcome evaluation, we assessed potential adverse effects including intraocular inflammation, retinal vasculitis, retinal pigment epithelial tear, severe vision loss (15 letters or more) due to any cause, procedure-related adverse effects such as traumatic cataract formation and retinal tear, as well as any systemic adverse effects reported by the patient.

### Statistical analysis

Wilcoxon’s signed-rank test was used to analyze changes in BCVA, CST, sf-CT, and injection interval. Dunn’s multiple comparison test was used to evaluate the baseline characteristics of patients enrolled in the study. One-way ANOVA and Pearson’s *t*-test were used to compare the clinical characteristics of patients who switched to IVF and those who continued IVBr. Repeat tests were not performed, so no correction was applied for multiple comparisons. All statistical analyses were performed using JMP Pro software version 17.0.0 (SAS Institute Inc., Cary, NC). A *P*-value of < 0.05 was considered statistically significant. Results are reported as the median [interquartile range].

## Results

### Patient demographics

The study included 43 men (71.6%), and the mean age was 75.5 [72.0–82.0] years (Table 1). Twenty-seven eyes had macular neovascularization (MNV) (type 1, n = 19; type 2, n = 6; type 3, n = 2) and 36 had polypoidal choroidal vasculopathy. The patients had received 25.0 [12.0–31.0] injections of other anti-VEGF agents and had been followed up 67.2 [47.2–100] months before their first IVF injection. Throughout the follow-up period, the patients were managed with a TAE regimen with a median treatment interval of 11 weeks, resulting in a well-controlled baseline BCVA of 0.222 [0.046–0.522], CST of 223 [193–267] µm, and sf-CT of 166 [115–242] µm. sf-CT was significantly greater in the dry macula group than in the IVF continuous group and switch-back group (*P* = 0.035 and 0.0058, respectively; see Supplementary Table S1).

**Table 1:** Patient demographic and clinical characteristics.

	All	IVF continuation group	Switch-back group	PDT combination group	Dry macula group
Eyes, n	63	40	16	3	4
Age, years [IQR]	75.5 [72.0–82.0]	76.5 [71.5–82.5]	75 [72–81.5]	76.5 [71–82]	75 [68–82.8]
Male sex, n (%)	43 (71.6%)	29 (76.3%)	10 (58.8%)	1 (50%)	3 (75%)
Axial length, mm [IQR]	23.7 [23.2–24.3]	23.3 [23.8–24.1]	23.7 [23.3–24.6]	24.9 [23.7–25.4]	23.9 [23.8–24.1]
Subtypes					
Type 1 MNV	19	13	4	0	2
Type 2 MNV	6	6	0	0	0
Type 3 MNV	2	1	1	0	0
PCV	36	20	11	3	2
Prior IVF					
LogMAR BCVA [IQR]	0.222 [0.046–0.522]	0.155 [0.046–0.528]	0.398 [0.155–0.655]	0.301 [0.046–0.398]	0.310 [0.128–0.492]
CST, µm [IQR]	223 [193–267]	223 [200–257]	233 [192–274]	452 [176–452]	190 [181–228]
sf-CT, µm [IQR]	166 [115–242]	161 [120–238]	147 [99–186]	257 [237–257]	329 [281–421]
No. of injections, [IQR]	25.0 [12.0–31.0]	26 [15.3–30.8]	24.0 [15.0–32.3]	6 [4.0–9.0]	19.0 [5.0–36.8]
Injection interval, weeks [IQR]	11.0 [9.0–13.0]	10 [9.0–13.0]	10.0 [8.0–14.5]	12.7 [8.0–13.0]	13.0 [12.0–14.8]
Disease duration, months [IQR]	67.2 [47.2–100]	67.1 [52.2–107]	75.8 [50.6–108]	39.1 [15.3–107]	58.9 [20.8–86.4]

IVF, intravitreal faricimab; PDT, photodynamic therapy; MNV, macular neovascularization; PCV, polypoidal choroidal vasculopathy; BCVA, best-corrected visual acuity; sf-CT, subfoveal choroidal thickness; CST, central subfield macular thickness; IVF, intravitreal faricimab; LogMAR, logarithm of the minimum angle of resolution; MNV, macular neovascularization; IQR, interquartile range.

### Changes in BCVA, CST, sf-CT, and number of injections per year

Table 2 shows the changes in BCVA, CST, sf-CT, and injection interval at 1 year after switching to IVF. BCVA was maintained over the 1-year follow-up period in both the IVF continuation group and switch-back group. In the switch-back group, there was a slight increase in CST (8 [− 25.5 to 40.0] µm) at the timing of switching back to IVBr, which returned to baseline levels at 1 year (see Supplementary Table S2). In contrast, the CST was maintained over the 1-year follow-up period in the IVF continuation group, sf-CT significantly decreased over 1 year in the IVF continuation group (− 19.5 [− 45.5 to 7.75] µm, *P* = 0.015) (Table 2). Changes in BCVA, CST, and sf-CT were not significantly different between the IVF continuation group and switch-back group (*P* = 0.87, 0.71, 0.17, respectively). There were no statically significant differences in the injection intervals before and after switching to IVF (Table 2).

**Table 2:** Changes in BCVA, CST, and sf-CT After the Initial IVF Injection by Subgroup.

Subgroup	IVF continuation group n = 40			Switch-back group n = 16		
	Baseline	A year after initial IVF	*P*-value	Baseline	A year after initial IVF	*P*-value
LogMAR BCVA [IQR]*	0.155 [0.046–0.528]	0.204 [0.059–0.398]	0.58	0.398 [0.155–0.655]	0.523 [0.155–0.761]	0.89
CST, µm [IQR]*	223 [200–257]	216 [188–237]	0.11	233 [192–274]	215 [174–262]	0.46
sf-CT, µm [IQR]*	161 [120–238]	142 [110–218]	**0.015**	147 [99–186]	136 [111–185]	0.90
Injection intervals, week [IQR]*	10 [9.0–13.0]	11.5 [9.3–14.0]	0.65	10.0 [8.0–14.5]	10 [8.3–12.0]^†^	0.77

Bold indicates statistical significance at *P* < 0.05.

BCVA, best-corrected visual acuity; sf-CT, central choroidal thickness; CST, central subfield macular thickness; IVF, intravitreal faricimab; LogMAR, logarithm of the minimum angle of resolution; IQR, interquartile range.

*Wilcoxon’s signed-rank test.

^†^One patient could pause injections and switch to a pro re nata regimen because no exudative change was observed for over 20 weeks, and this patient was excluded from this result.

On average, the patients in the IVF continuation group received 5 [4–6] IVF injections over 1 year while patients who paused injections received 2.5 [2.0–3.8] IVF injections. Patients who were switched back received 4 [2.8–4.5] IVBr injections after receiving 2 [1.3–3.7] IVF injections. Patients who received PDT received 7.0 [6.0–7.0] IVF injections (Table 3). The injection interval of IVF and IVBr at 1 year was not significantly different between the IVF continuation group and switch-back group (11.5 vs 11.0 weeks, respectively, *P* = 0.64).

**Table 3:** Patient demographic and anatomical outcomes at 1 year after the initial IVF injection.

Eyes, n	IVF continuation group	Switch-back group	PDT combination group	Dry macula group
	40	16	3	4
LogMAR BCVA [IQR]	0.204 [0.059–0.398]	0.412 [0.155–0.655]	0.301 [− 0.079 to 0.523]	0.310 [0.128–0.492]
CST, µm [IQR]	216 [188–237]	211 [172–245]	248 [141–325]	181 [170–227]
sf-CT, µm [IQR]	142 [110–218]	134 [108–190]	225 [178–229]	313 [274–387]
No. of injections, [IQR]	5 [4–6]	2[1.3–3.7], 4 [2.5–4.5]*	7.0 [6.0–7.0]	2.5 [2.0–3.8]
Injection interval, weeks [IQR]	11.5 [9.3–14.0]	11 [8.3–12.9]^†^	12 [8–16]^‡^	NA

IVF, intravitreal faricimab; LogMAR, logarithm of the minimum angle of resolution; BCVA, best-corrected visual acuity; sf-CT, central choroidal thickness; CST, central subfield macular thickness; IQR, interquartile range.

*Number of faricimab and brolucizumab injections are shown, respectively.

^†^One patient could pause injections and switch to a pro re nata regimen because no exudative change was observed for over 20 weeks, and this patient was excluded from this result.

^‡^Two eyes from a patient were monitored without treatment because no exudative change was observed for over 8 weeks, and this patient is excluded from this result.

### Changes in injection intervals before and after switching

When patients were classified into two groups based on whether the pre-switch injection interval was within 12 weeks or 12 weeks or more over, there was no statistical difference between groups. (See Supplemental Table S3) Dry macula group comprises patients with no disease activity for 20 weeks or more. They were treated as if they reached a maximun dosing interval of 20 weeks to prevent biased conclusions. In eyes that had an injection interval of less than 12 weeks before switching, the IVF injection interval was longer in the IVF continuation group and the switch-back group 1 year after the first IVF (2.0 and 3.0 weeks [*P* = 0.0007, 0.0078], respectively), whereas in eyes of the switch-back group that had an injection interval of 12 weeks or more before switching, the injection interval decreased (− 4.8 weeks [*P* = 0.031]). (Table 4).

**Table 4:** Sub-analysis of changes in BCVA, CST, CCT, and injection interval, stratified by injection interval ≤ 12 versus > 12 weeks before switching.

	Within 12 weeks		Over 12 weeks	
	IVF continuation group and dry macula group (N = 28 + 2)	Switch-back group (N = 10)	IVF continuation group and dry macula group (N = 12 + 2)	Switch-back group (N = 6)
	Difference (*P*-value)	Difference (*P*-value)	Difference (*P*-value)	Difference (*P*-value)
LogMAR BCVA*	0.0 (0.70)	0.048 (0.54)	− 0.028 (0.98)	− 0.040 (0.53)
CST, µm*	0.0 (0.72)	− 19.0 (0.082)	− 8.5 (0.10)	33 (0.69)
sf-CT, µm*	− 14.5 (0.21)	− 6.5 (0.82)	− 27.5 (0.0031)	9 (0.69)
Injection interval, weeks*	2.0^†^ (0.0007)	3.0 (0.0078)	− 2.5^†^ (0.10)	− 4.8 (0.031)
No. IVF injections	5 [4–6]	2 [1–3.3]^‡^	4 [4, 5]	2.5 [1.8–4]^‡^

Bold indicates statistical significance at *P* < 0.05.

BCVA, best-corrected visual acuity; sf-CT, central choroidal thickness; CST, central subfield macular thickness; IVF, intravitreal faricimab; LogMAR, logarithm of the minimum angle of resolution; IQR, interquartile range.

*Wilcoxon’s signed-rank test, ^†^dry macula group is set to 20 weeks, ^‡^number of IVF injection before switch-back.

### Safety outcomes

**Figure 1:**
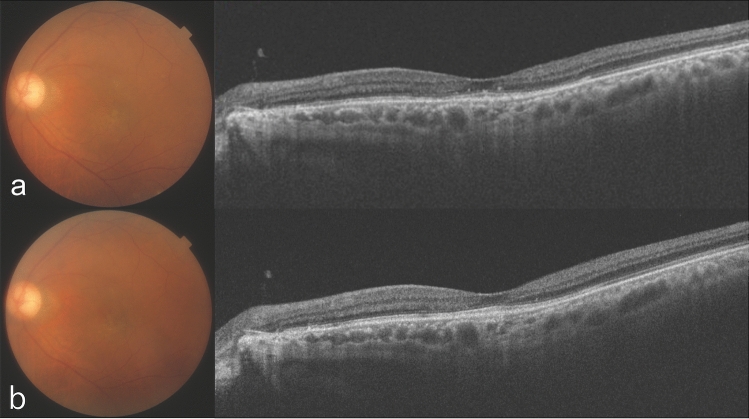
A 76-year-old man who had received 4 intravitreal brolucizumab injections and an intravitreal aflibercept injection before the initial intravitreal faricimab (IVF) injection. (**a**) Optical coherence tomography (OCT) shows a thick choroid and dilated large choroidal vessels underlying an attenuated choriocapillaris with no exudative change at the initial IVF. The injection interval was 12 weeks before the last intravitreal brolucizumab injection. (**b**) OCT shows no exudative change a year after the initial IVF injection.

**Figure 2:**
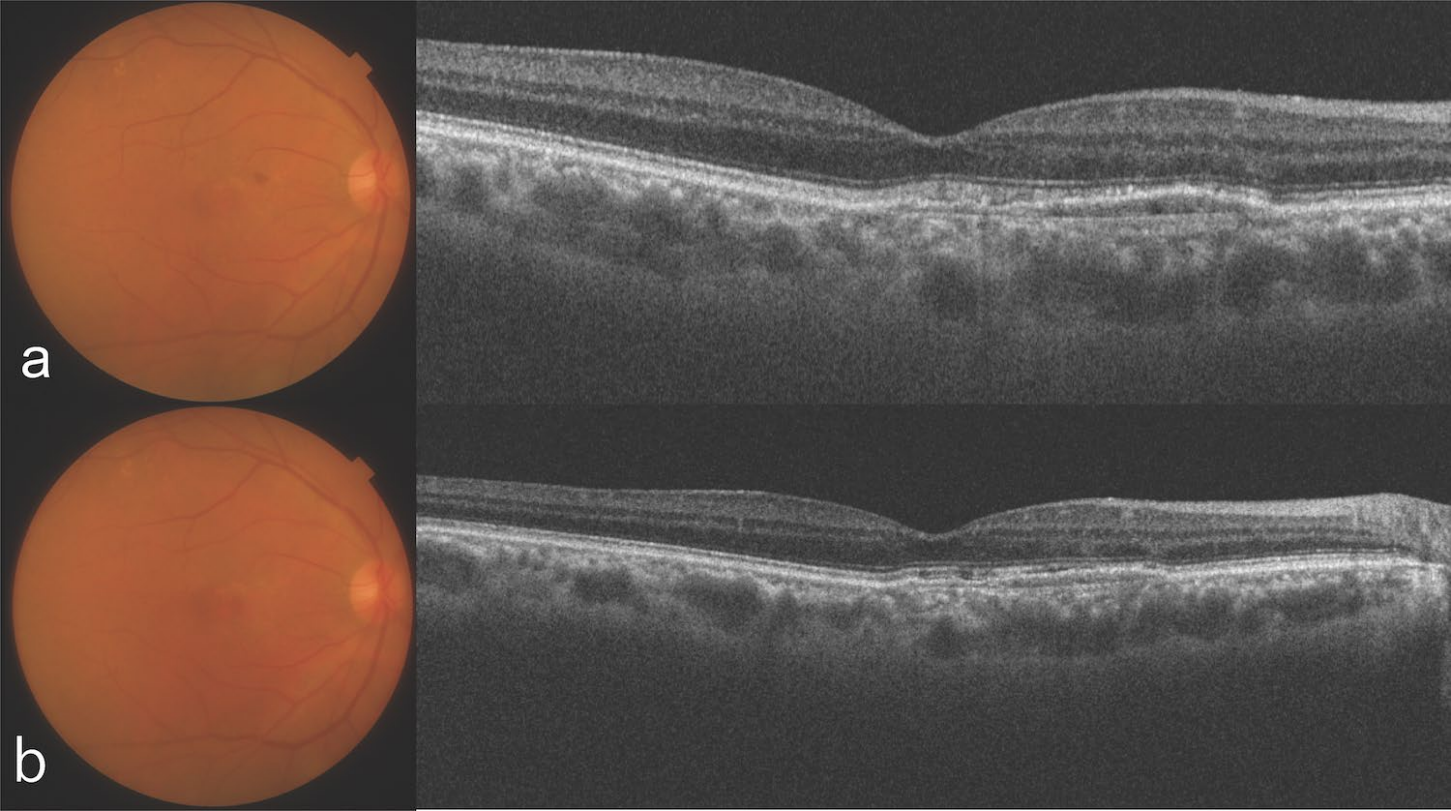
A 74-year-old man who had received 7 intravitreal brolucizumab injections and 26 intravitreal aflibercept injections before the initial intravitreal faricimab (IVF) injection. (**a**) Optical coherence tomography (OCT) shows a thick choroid and dilated large choroidal vessels underlying an attenuated choriocapillaris with retinal pigment epithelium detachment at the initial IVF. A fundus image shows subretinal hemorrhage. The injection interval was 14 weeks before the last intravitreal brolucizumab injection. (**b**) OCT shows no exudative change a year after initial IVF.

**Figure 3:**
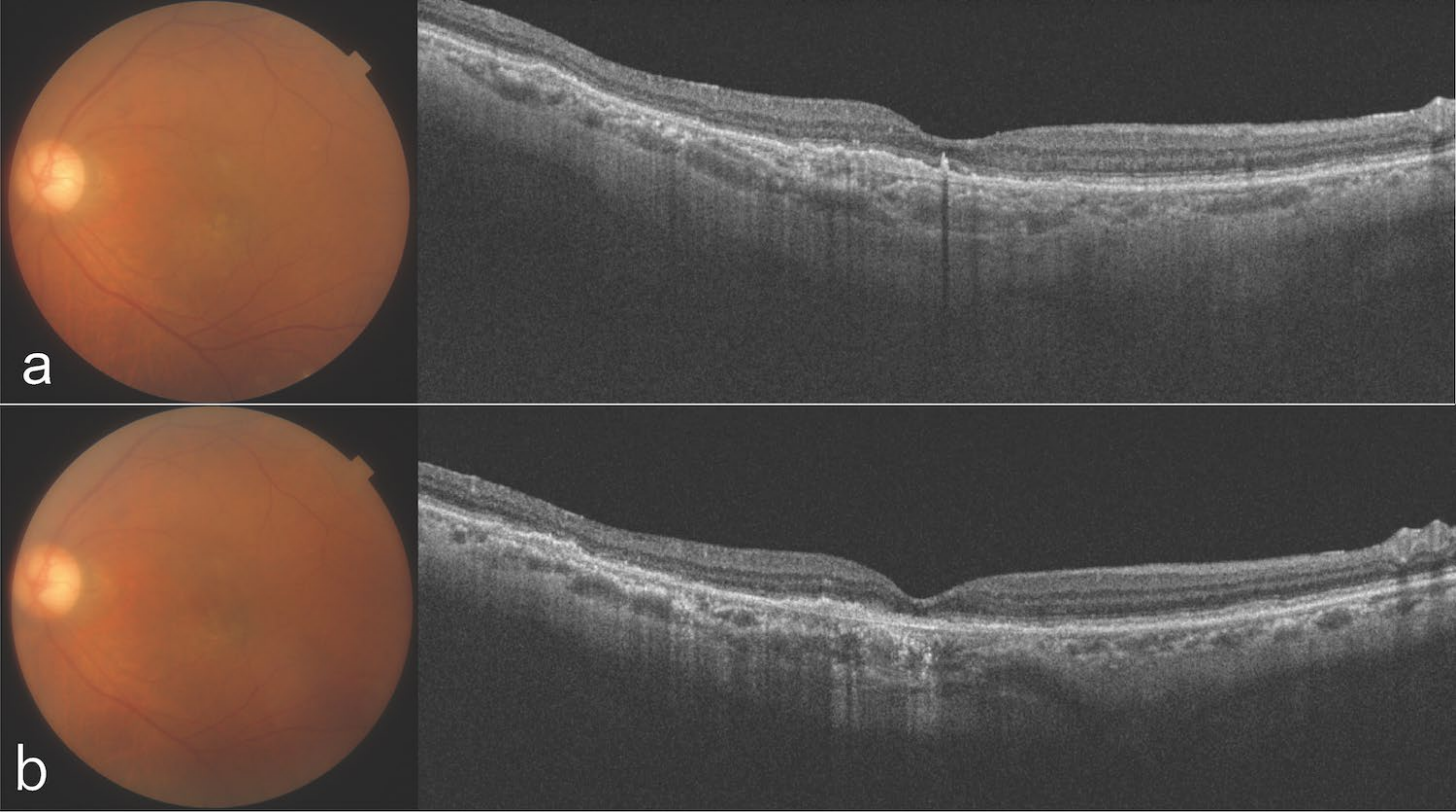
An 86-year-old woman who had received 8 intravitreal brolucizumab injections and 30 intravitreal aflibercept before the initial intravitreal faricimab (IVF) injection. (**a**) Optical coherence tomography (OCT) shows a thick choroid and dilated large choroidal vessels underlying an attenuated choriocapillaris with no exudative change at the initial IVF injection. The injection interval was 15 weeks before the last intravitreal brolucizumab. (**b**) OCT shows no exudative change or dilated choroidal vessels a year after initial IVF.

**Figure 4:**
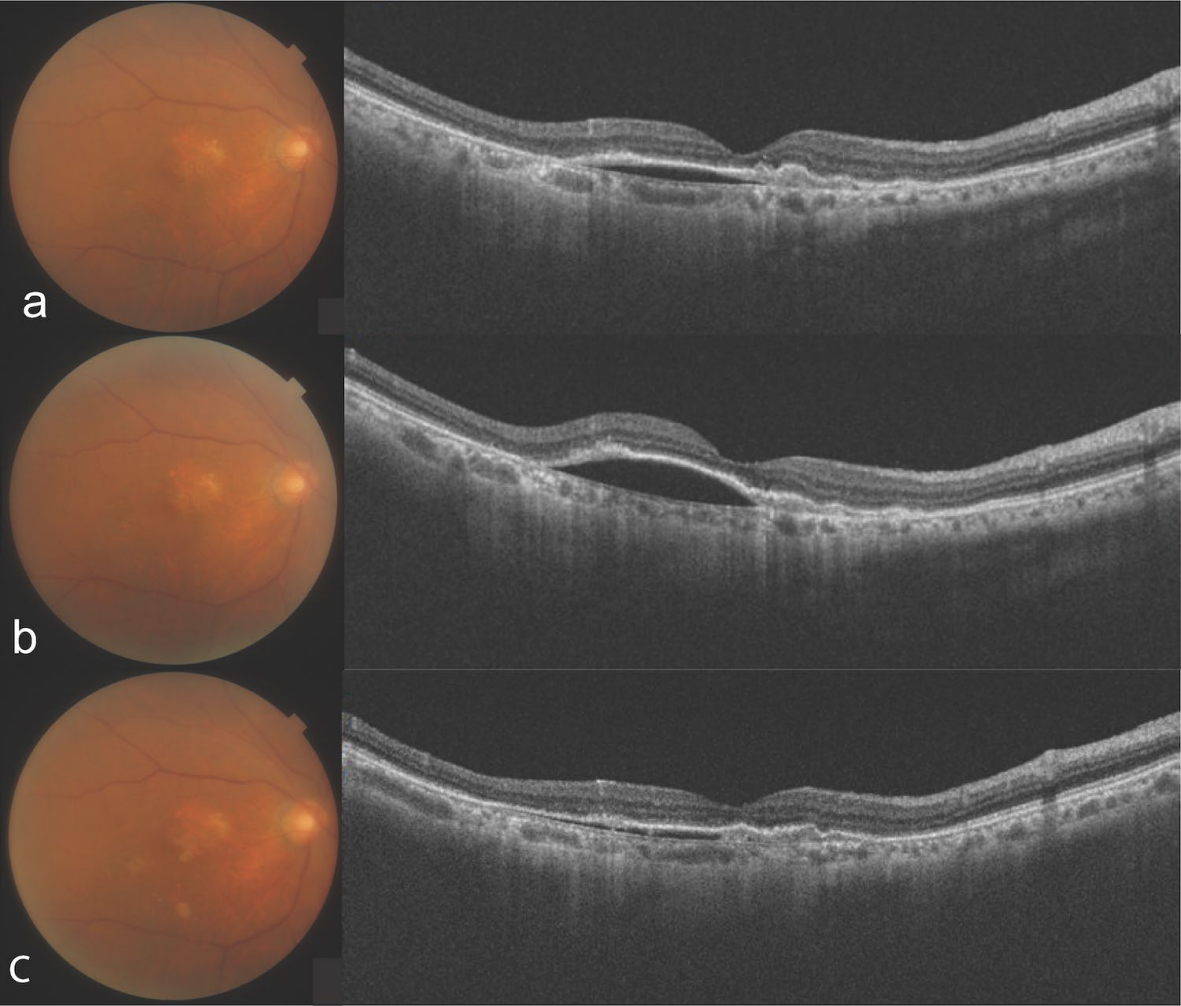
An 81-year-old woman who had received 3 intravitreal brolucizumab injections (IVBr) and an intravitreal aflibercept before the initial intravitreal faricimab (IVF) injection. (**a**) Optical coherence tomography (OCT) shows a relatively thin choroid and undilated choroidal vessels with infant intraretinal fluid and serous retinal pigment epithelium detachment (PED) at the initial IVF. (**b**) After the initial IVF. OCT shows PED increased. There was an increase in the PED height. Following a discussion with the patient, the decision was made to switch back to IVBr. (**c**) A year after the initial IVF. OCT shows low and wide serous PED. She received 6 IVBr after the initial IVF.

Representative images for the dry macula and switch-back group are shown in Figs. 1, 2, 3 and 4, respectively. In the safety outcome evaluation, we did not observe any adverse effects including intraocular inflammation, retinal vasculitis, retinal pigment epithelial tear, severe vision loss, procedure related adverse effects such as traumatic cataract formation and retinal tear, and no systemic adverse effects were reported by patients.

## Discussion

This retrospective study investigated real-world 1-year outcomes of nAMD patients who switched from IVBr to IVF. The results showed that 40 eyes of 38 patients (63.3%) were treated with IVF for a year after switching from IVBr, and 4 eyes of 4 patients (6.7%) could pause injections. Although 16 eyes of 16 patients (26.7%) were switched back to IVBr, changes in BCVA, CST, and sf-CT were not significantly different between the IVF continuation group and switch-back group.

Treatment could be paused in 4 eyes of 4 patients (6.7%) after 2.5 [2.0–3.8] IVF injections (dry macula group). The baseline characteristics were not significantly different in these patients compared with the other groups, except for sf-CT, which was significantly thicker in the dry macula group than in the IVF continuation group and switch-back group. Previous studies have shown that Ang-2 is higher in eyes with pachychoroid features^31^, that a significant proportion of patients with nAMD have pachychoroid features in Asian countries, and that the phenotype of nAMD is known to differ from between Asian and Caucasian patients^32–35^. An analysis of Japanese and Asian subgroups showed favorable results in terms of injection interval in the pooled global TENAYA/LUCENE trials^36,37^. Thus, dual Ang-2/VEGF-A inhibition might potentiate good efficacy for patients with pachychoroid features. Indeed, in the 4 eyes for which injections were paused, OCT showed not only a thick choroid but also dilated large choroidal vessels underlying an attenuated choriocapillaris, which are saliant features of pachychoroid disorders. Furthermore, our previous report of a single IVF injection in eyes with nAMD which were previously treated with IVBr showed the reduction in sf-CT^17^. Sf-CT decreased in patients who were switched to IVF and maintained on treatment for 1 year. The biological significance of Ang-2 has been experimentally demonstrated in terms of the effect of Tie2 activation on choroidal ischemia^38^. Additionally, knockout mice of Angptl1 in choroidal vessels have been shown to develop “pachychoroid (thick choroid)”^39^. Therefore, it is conceivable that Ang2 inhibition can improve choroidal circulation and ameliorate the morbidity caused by thickened choroidal thickness, further studies are needed to envelope the detailed mechanism.

Although the injection intervals of IVF and IVBr at 1 year were not significantly different between the IVF continuation group and the switch-back group, when compared according to injection interval before switching to IVF, eyes that had an injection interval of less than 12 weeks before switching showed an extension of the injection interval. Brolucizumab has been shown to have a relatively potent anti-exudative effect^4–7^, but in cases with recurrence within 12 weeks, it may be beneficial to consider the suppression of Ang-2 activity. The underlying cause of this change is unclear, and further studies are needed.

Because faricimab is not permitted to be administered at intervals shorter than 8 weeks in Japan during maintenance phase, this restriction might have forced patients to switch back to brolucizumab or resort to rescue therapy with PDT. This limitation would have influenced the decision-making process of patients and could explain why some individuals opted to switch back to brolucizumab despite the comparable efficacy between the treatments. In the present study, 19 eyes (31.7%) did not continue using IVF and were switched back to IVBr with or without PDT; however, BCVA was not worse when switching back visit compared with baseline, while CST was slightly increased. In such cases, shortening of the intervals between the injections should be another option. We speculate that this switching strategy may also have influenced why patients quickly switched back to brolucizumab after only a few injections. To assess the impact of the initial IVF injections, patients were advised to maintain their regular visit intervals (± 1 week) regardless of exudative changes on that day. Our hypothesis was that if the previous anti-VEGF agent had a similar effect to faricimab, there would be no significant differences in exudative changes and choroidal thickness. Additionally, we predicted that stronger effectiveness would lead to better suppression of exudation and choroidal thickness, while weaker effectiveness would result in increased changes in both of these parameters. Consequently, patients might perceive brolucizumab to be more effective if they experienced increased CST thickening on faricimab and chose to switch back to it^17^. Ultimately, however, the increase in CST thickness was only minor (8 [− 25.5 to 40.0] µm). As such, investigating the efficacy of IVF without switching back to brolucizumab would be valuable in future studies.

This study has some limitations. First, all the patients were Japanese, which may limit the generalizability of the findings to other ethnic populations. The impact of ethnic and genetic variations on treatment responses and outcomes cannot be fully elucidated based on this study alone. Second, in Japan, faricimab is not permitted to be administered at intervals shorter than 8 weeks at maintenance phase. This restriction might have forced patients to switch back to brolucizumab or resort to rescue therapy with PDT. Thirdly, the sample size was relatively small. Although there was no statistical difference in the treatment intervals before and after switching to IVF, this might be due to the small sample size. A larger sample size would enhance the generalizability of the results. Although a reduction in sf-CT was observed in eyes switched from brolucizumab to faricimab, and the sf-CT was significantly thicker in the dry macula group than in the IVF continuation and switch-back groups, we were not able to adjust changes in sf-CT for age and axial length. The clinical significance of this change is unknown and remains a subject for further investigation. Finally, in connection with this being a retrospective real-world analysis, treatment decisions were made in a non-randomized, patient-specific manner. This study did not directly compare the relative efficacy of brolucizumab versus faricimab. Long-term real-world follow-up data will be crucial for understanding the usefulness of IVF in the future.

In conclusion, this retrospective study reported the real-world outcomes of nAMD patients switched from IVBr to IVF. The findings suggest that faricimab has a favorable safety profile and satisfactory effectiveness, making it a promising alternative to brolucizumab in the treatment of nAMD. A major limitation of faricimab is the current restriction on its use in Japan, where it is not permitted to be administered at intervals shorter than 8 weeks, thus requiring a switch back to brolucizumab or rescue therapy with PDT.

### Supplementary Information

Supplementary Tables.

## Data Availability

The datasets used and/or analysed during the current study are available from the corresponding author on reasonable request.
