# Supplementary material for: One-year visual and anatomical outcomes of intravitreal faricimab injection for neovascular age-related macular degeneration after prior brolucizumab treatment
Source: Sci Rep. 2024 Apr 20;14:9087. doi: 10.1038/s41598-024-59894-8 (PMC11032343; doi:10.1038/s41598-024-59894-8)
Supplement: Supplementary file 1 — Supplementary Tables. [file 41598_2024_59894_MOESM1_ESM.docx]

**Supplementary Information for**

**One-Year Visual and Anatomical Outcomes of Intravitreal Faricimab Injection for Neovascular Age-Related Macular Degeneration After Prior Brolucizumab Treatment**

Hironori Takahashi1, M.D., Satoru Inoda1, M.D., Ph.D., Hidenori Takahashi1, M.D., Ph.D., Ryota Takahashi1, M.D., Yuto Hashimoto1, M.D., Hana Yoshida1, M.D., Hidetoshi Kawashima1, M.D., Ph.D., Yasuo Yanagi2,3, M.D., Ph.D

**Contents**

Supplementary Tables S1. Baseline Characteristics Comparison

Supplementary Tables S2. Changes in BCVA, CST, and CCT Before and After the Visit of Switching Back

Supplementary Tables S3. Baseline characteristics by Injection Interval Before switching

Supplementary Tables S4. Patient Demographic and Clinical Characteristics

Supplementary Table S1. Baseline Characteristics Comparison

| Baseline BCVA, (LogMAR) | IVF continuation group | | Switch-back group | | PDT combination group | |  |
| --- | --- | --- | --- | --- | --- | --- | --- |
|  | Mean difference | P-value | Mean difference | P-value | Mean difference | P-value |  |
| Switch-back group | −8.94 | 0.59 |  |  |  |  |  |
| PDT combination group | −0.69 | 0.99 | −7.92 | 0.99 |  |  |  |
| Dry macula group | 5.81 | 0.99 | −2.88 | 0.99 | 4.79 | 0.99 |  |
| Baseline CST, µm | **IVF continuation group** | | **Switch-back group** | | **PDT combination group** | |  |
|  | Mean difference | P-value | Mean difference | P-value | Mean difference | P-value |  |
| Switch-back group | −0.68 | 0.99 |  |  |  |  |  |
| PDT combination group | −11.0 | 0.99 | 10.3 | 0.99 |  |  |  |
| Dry macula group | −13.5 | 0.96 | −14.2 | 0.99 | −24.5 | 0.47 |  |
| Baseline CCT, µm | **IVF continuation group** | | **Switch-back group** | | **PDT combination group** | |  |
|  | Mean difference | P-value | Mean difference | P-value | Mean difference | P-value |  |
| Switch-back group | 7.3 | 0.99 |  |  |  |  |  |
| PDT combination group | −17.0 | 0.73 | 24.3 | 0.21 |  |  |  |
| Dry macula group | 26.5 | **0.035** | 33.8 | **0.0058** | 9.2 | 0.99 |  |
| Axial length, µm | **IVF continuation group** | | **Switch-back group** | | **PDT combination group** | |  |
|  | Mean difference | P-value | Mean difference | P-value | Mean difference | P-value |  |
| Switch-back group | −1.6 | 0.99 |  |  |  |  |  |
| PDT combination group | −17.3 | 0.68 | 15.6 | 0.99 |  |  |  |
| Dry macula group | 6.4 | 0.99 | 4.7 | 0.99 | −10.7 | 0.99 |  |
| No. of injection | **IVF continuation group** | | **Switch-back group** | | **PDT combination group** | |  |
|  | Mean difference | P-value | Mean difference | P-value | Mean difference | P-value |  |
| Switch-back group | 0.28 | 0.99 |  |  |  |  |  |
| PDT combination group | 27.5 | 0.07 | −27.1 | 0.11 |  |  |  |
| Dry macula group | −3.6 | 0.99 | −3.3 | 0.99 | 23.6 | 0.55 |  |
| Injection interval, week | **IVF continuation group** | | **Switch-back group** | | **PDT combination group** | |  |
|  | Mean difference | P-value | Mean difference | P-value | Mean difference | P-value |  |
| Switch-back group | 3.2 | 0.99 |  |  |  |  |  |
| PDT combination group | −0.5 | 0.99 | 3.8 | 0.99 |  |  |  |
| Dry macula group | 12.6 | 0.99 | 15.9 | 0.71 | 11.8 | 0.99 |  |
| Disease duration, month | | **IVF continuation group** | | **Switch-back group** | | **PDT combination group** | |
|  |  | Mean difference | P-value | Mean difference | P-value | Mean difference | P-value |
| Switch-back group | | -1.8 | 0.99 |  |  |  |  |
| PDT combination group | | 11.4 | 0.99 | -13.2 | 0.99 |  |  |
| Dry macula group | | -8.0 | 0.99 | -9.8 | 0.99 | 3.2 | 0.99 |

Dunn’s multiple comparisons test.

Bold indicates statistical significance at P < 0.05.

BCVA, best-corrected visual acuity; CCT, central choroidal thickness; CST, central subfield macular thickness; IVF, intravitreal faricimab; LogMAR, logarithm of the minimum angle of resolution; IQR, interquartile range; PDT, photodynamic therapy.

Supplementary Table S2. Changes in BCVA, CST, and CCT Before and After the Visit of Switching Back

|  | **Before switching back** | **After switching back** | **Difference (P-value)** |
| --- | --- | --- | --- |
| **LogMAR BCVA [IQR]^*^** | 0.372 [0.216–0.699] | 0.372 [0.222–0.699] | 0.00 [−0.040 to 0.017] (0.30) |
| **CST, µm [IQR]^*^** | 250 [178–321] | 258 [169–367] | 8 [−25.5 to 40.0] (0.11) |
| **CCT, µm [IQR]^*^** | 146 [103–189] | 149 [100–202] | 0 [−11.0 to 3.5] (0.84) |

*Wilcoxon’s signed-rank test.

BCVA, best-corrected visual acuity; CCT, central choroidal thickness; CST, central subfield macular thickness; IVF, intravitreal faricimab; LogMAR, logarithm of the minimum angle of resolution; IQR, interquartile range.

Supplementary Table S3. Baseline characteristics by Injection Interval Before switching

|  | |  | **Within 12 weeks** | | **Over 12 weeks** | | |
| --- | --- | --- | --- | --- | --- | --- | --- |
|  | |  | **IVF continuation group and dry macular group**  **(N = 28+2)** | **Switch-back group**  **(N = 10)** | | **IVF continuation group and dry macular group (N = 12+2)** | **Switch-back group**  **(N = 6)** |
| **Eyes, n** | | | 30 | 10 | | 14 | 6 |
| **Age, years [IQR]*** | | | 78 [69.5-82.5] | 74 [70.8-77.8] | | 75.5 [74.0-84.3] | 78.5 [72.0-84.5] |
| **Male sex, n (%)*** | | | 22 (73.3%) | 6 (60.0%) | | 10 (71.4%) | 4(66.7%) |
| **Axial length, mm [IQR]*** | | | 23.8 [23.3-24.5] | 23.7 [22.7-24.6] | | 23.4 [23.0-24.0] | 23.7 [23.4-24.9] |
| **Subtypes,*,^†^** | | | 11, 4, 0, 15 | 4, 0, 0, 6 | | 4, 2, 1, 7 | 0, 0, 1, 5 |
| **Prior IVF*** | **LogMAR BCVA [IQR]** | | 0.125 [0.0458-0.429] | 0.350 [0.128-0.473] | | 0.222 [0.0727-0.567] | 0.522 [0.116-1.34] |
|  | **CST, µm [IQR]** | | 217 [192-241] | 233 [185-280] | | 231 [201-263] | 231 [192-290] |
|  | **CCT, µm [IQR]** | | 153 [114-274] | 163 [105-192] | | 174 [154-315] | 117 [54-201] |
|  | **No. of injections, [IQR]** | | 26.0 [11.8-31.3] | 25.5 [13.5-34.8] | | 25.5 [15.8-28.3] | 21.5 [13.3-28.8] |
|  | **Injection interval, weeks [IQR]** | | 10.0 [8.21-12.0] | 8.0 [6.7-9.5] | | 15.0 [13.0-16.3] | 15.5 [13.0-16.0] |
|  | **Disease duration, months [IQR]** | | 65.1 [46.3-92.0] | 75.8 [43.0-113] | | 77.1 [51.2-124.7] | 76.1 [51.1-106] |

*Kruskal-Wallis test, **^†^**type 1 MNV, type 2 MNV, type 3 MNV, and polypoidal choroidal vasculopathy

BCVA, best-corrected visual acuity; CCT, central choroidal thickness; CST, central subfield macular thickness; IVF, intravitreal faricimab; LogMAR, logarithm of the minimum angle of resolution; IQR, interquartile range.

Supplementary Table S4. Patient Demographic and Clinical Characteristics

|  | **Patients who selected IVF**  **N=93** | **Patients who continued IVBr**  **N=15** | **P-value** |
| --- | --- | --- | --- |
| **Age, y (SD) *** | 76.1 (8.8) | 76.7 (7.2) | 0.79 |
| **Sex, male (%)†** | 69 (74.2) | 10 (66.7) | 0.37 |
| **AXL, mm (SD) †** | 23.7 (1.1) | 24.1 (1.2) | 0.20 |
| **Subtypes‡** | 25, 11, 2, 55 | 8, 0, 0, 7 | 0.14 |
| **LogMAR BCVA (SD) *** | 0.283 (0.392) | 0.301 (0.234) | 0.86 |
| **Injection intervals, week (SD) *** | 11.7 (2.9) | 14.0 (3.1) | **0.0072** |
| **Disease duration, month (SD) *** | 52.9 (30.7) | 43.2 (30.5) | 0.26 |

*One-way ANOVA; † Pearson’s *t*-test.

‡Subtypes: type 1 MNV, type 2 MNV, type 3 MNV, polypoidal choroidal vasculopathy.

Bold indicates statistical significance at P < 0.05.

IVF, intravitreal faricimab; IVBr, intravitreal brolucizumab; AXL, axial length; LogMAR, logarithm of the minimum angle of resolution; BCVA, best-corrected visual acuity; MNV, macular neovascularization.
